# Hospital News

**Published:** 1922-04

**Authors:** 


### Hospital News.

The new Wincanton Hospital, which was formerly a convent, has been opened by Lady Theodora Guest.

The Committee of Lowestoft Hospital, which celebrates its centenary this year, hopes shortly to have " good, comfortable paying wards," and eventually a general extension.

Mr. Tom Ralph, hon. secretary of the Slough Hospital Subscription Committee, is retiring so soon as a successor can be found, because he is unable to devote so much time to the work as hitherto.

Trowbridge Cottage Hospital is reported to have joined the voluntary hospitals association, which we presume to mean the appropriate Regional Committee of the British Hospitals Association.

Mr. Charles Lyle, M.P., president of Queen Mary's Hospital, Stratford, has laid the foundation stone of the new Maternity Home, towards the cost of which he has given £10,000 in memory of his wife.

The Purley and District War Memorial Hospital, built at a cost of £1,200, was opened in March by Major-General E. D. Swinton, C.B., D.S.O. It forms the extension to the Purley Cottage Hospital.

The board of the Royal Hospital, Sheffield, has lately called in an expert to inspect the food, catering and kitchen. His report states that there is no waste in stuff or materials, and that the catering is efficient.

Colonel R. W. Williams-Wynn, Chairman of the Alexandra Hospital, Rhyl, says that there are many vacancies in the winter when the hospital can accommodate children whom it is obliged to refuse in the summer rush.

Women have now been appointed to serve on the committee of management of the Liverpool Hospital for Women, which hitherto allowed only a woman president and vice-president to attend the meetings of its general committee.

The Earl of Onslow, Chairman of the Voluntary Hospitals Commission, has announced that in all counties except two the new Local Committees to assist in hospital co-ordination and in the distribution of the Government grant of £500,000 have been set up.

Mr. Walter Hanson, who has been secretary of the publicity and other committees of the Sheffield Royal Infirmary Workers' Committee, has been presented with a silver rose-bowl in recognition of his long service. Since 1913 Mr. Hanson has collected £3,000.

Miss Frances Ivens, M.B., M.S., has proposed to the Liverpool Branch of the National Council of Women of Great Britain and Ireland a Women's Hospital Guild to help the Liverpool hospitals. The project was welcomed. Its object is not only to raise money but to increase the number of women on hospital boards and to encourage better provision for maternity cases.

Important extensions, estimated at £50,000 last December, are about to be carried out at the Chesterfield and North Derbyshire Hospital. A sum of over £40,000, or four-fifths of the estimated total, has been raised. The extensions include a new out-patient department, a laundry, and the re-housing of the nursing, domestic, and administrative staff. The new out-patient department, the chief item, is expected to be ready during the year.

The Historic Monuments Committee of St. Andrew's Society (Glasgow) has appealed very strongly to the Glasgow Royal Infirmary to rescind its decision to demolish the famous Lister Ward at the hospital.

The Physician Superintendent of the Aberdeen Royal Infirmary states in his annual report that the religious revival which occurred in the towns and fishing villages of the north-east of Scotland at the end of last year, contrary to general belief, was not responsible for the illness of any of the patients admitted to his institution.

A brief history of the Royal Mineral Water Hospital, Bath, which was founded by Beau Nash and Dr. Oliver in 1739, has been compiled and can be obtained free from the Registrar. The old Act of 1597, which gave to the diseased and impotent poor the right to free use of the Baths, has its analogue in the custom of modern Unions, many of whom still send their poor cripples for treatment. It is stated that few of these Unions recognise the cost of the work which the hospital does for them. The history has a preface by Mr. Frederick Harrison.

We are glad to see the announcement of what promises to be another hospital history. This is " a record " of the Royal Devon and Exeter Hospital which Mr. J. Delpratt Harris has prepared. Even when these histories are modest in scope they are of great value, as the storehouse of the tradition in which a voluntary hospital should live. We have always defended portraits in the board room, and what are known as æsthetic memorials for the same reason. A hospital of any age without a written history has been incompletely served; and the Royal Devon and Exeter Hospital is 180 years old.

Mr. Edward Watson, honorary secretary of the Gateshead Children's Hospital, has been presented with a silver salver and a portrait of himself by the president, Lord Northbourne, the committee, the medical staff, and the matron, Miss Dawson. Mr. Watson is retiring from the secretaryship which he has held for 30 years. The hospital is only 35 years old, so Mr. Watson has watched its progress almost from the start. The principal change which he helped to bring about was the construction of the new wing in 1908. This gave the hospital in all 42 beds. Mr. Dawson has been elected a Vice-President. His assistant secretary is Mr. Hugh C. Watson, A.C.A.

The nurses employed by the Skipton Board of Guardians have found an advocate in the chairman of the house committee, Mr. G. Bottomley. He has been trying to prevail upon the Guardians to convert, at a cost of £150, the old infirmary into a Nurses' Home. During the recent influenza epidemic, Mr. Bottomley remarked, sick nurses had to sleep four in a room. The institution was crowded, and the nurses overworked. Another speaker remarked that the nurses had to climb 49 steps eight times a day to their bedrooms, and to traverse passages which some of the board had never seen. An " economist " declared that the old infirmary was useless, and had proved useless for every scheme suggested.

The Governors of the West End Hospital for Nervous Diseases are most anxious that their Hospital shall be of the greatest possible use to the community. Proposals are already maturing for the arrangement of a definite teaching programme and for the provision of suitable accommodation for laboratory and clinical research. Many general improvements are needed in the nursing quarters, and, as a commencement, extra houses have been rented in St. Katherine's precincts, in the near vicinity of the Hospital. Despite the success of the Festival Dinner, the Hospital is faced with a considerable deficiency, but it is hoped that a large sum of new money will be raised this year as the result of a combined scheme for obtaining organised collections from workpeople and employers.

